# The impact of effective communication skills training on the status of marital burnout among married women

**DOI:** 10.1186/s12905-021-01372-8

**Published:** 2021-06-03

**Authors:** Alireza Jafari, Ali Alami, Elham Charoghchian, Ali Delshad Noghabi, Mahbobeh Nejatian

**Affiliations:** 1 Social Determinants of Health Research Center, Gonabad University of Medical Sciences, Gonabad, Iran; 2 Department of Epidemiology and Bio-Statistics, School of Public Health, Social Determinants of Health Research Center, Gonabad University of Medical Sciences, Gonabad, Iran; 3 Student Research Committee, Mashhad University of Medical Sciences, Mashhad, Iran

**Keywords:** Marital boredom, Effective communication skills, Education, Intervention, Couple burnout

## Abstract

**Background:**

This study aimed to determine the impact of effective communication skills training intervention on the marital burnout among married women referring to health centers.

**Methods:**

In this quasi-experimental study, 94 participants were selected from a descriptive study from among 936 married women referring to health centers who had a high rate of marital burnout and were randomly divided to the experimental group (n = 47) and control group (n = 47). The educational intervention was designed and performed in 7 sessions of 45 min for the experimental group. In the two stages before and after the intervention, the demographic sections, the Pines Marital Burnout Scale and effective communication skills were used to collect data. Data were analyzed using SPSS software version 24 and inferential test of Chi-square, Paired sample t-test and Wilcoxon test.

**Results:**

There was no significant difference between the two groups before the intervention (*p* > 0.05). Performing the intervention in the experimental group significantly reduced the mean score of total marital burnout from 60.51 (± 14.96) to 51.82 (± 11.90), and reduced the mean score of marital burnout subscales, including physical, mental and emotional burnout. Also, in this study, the educational intervention of the experimental group significantly improved effective communication skills, and the mean score of effective communication skills increased from 85.12 (± 15.86) to 97.95 (± 14.53) (*p* < 0.001).

**Conclusion:**

Based on the positive impact of effective communication skills on reducing marital burnout, it is recommended that more attention should be paid to enhancing these skills in spouses and pre-marital programs.

**Supplementary Information:**

The online version contains supplementary material available at 10.1186/s12905-021-01372-8.

## Background

The history of marriage can be traced back to the beginning of history. Throughout history, marriage has been one of the most exciting and stressful events in everyone's life [1, 2]. The formation of a healthy marriage is one of the factors for the successful establishment of a family, which will undoubtedly have a positive impact on society [3]. But as time passed, problems appeared in the relationship between husband and wife, which reduced the love and affection between them and reduced life satisfaction [4]. One of the most important of these problems is marital burnout, which leads to emotional divorce and formal divorce among couples, and as a result, creates many problems for the couple's children and relatives [5, 6]. Marital burnout is one of these psychological disorders. Over time, they will reduce the love and relationship between couples, cause psychological problems, and lead to emotional divorce and formal divorce [1]. Marital burnout is caused by a mismatch between the facts and expectations of the couple, and its severity depends on the compatibility of the couple and their beliefs. The first article on the marital burnout was published in scientific journal in the mid-1970s. Pines was the first to extend burnout to non-work areas and other aspects of life, and was the first to introduce marital burnout [7].

Marital burnout refers to a painful state of physical, emotional, and psychological exhaustion that occurs when couples realize that despite all their efforts, a relationship does not and will not give meaning to their lives [8]. The onset of burnout is rarely sudden and is usually gradual. Maslach and Jackson believe that marital burnout is divided into three stages, including physical, emotional, and psychological exhaustion [9]. Physical burnout are characterized by symptoms such as fatigue, lethargy, chronic headaches, abdominal pain, sleep disturbances, loss of appetite, and overeating. Emotional burnout refers to feelings of resentment, unwillingness to solve problems, frustration, sadness, feelings of emptiness, feelings of lack of motivation, being trapped, absurdity, emotional turmoil, and even suicidal motives. Psychological burnout refers to low self-esteem, negative attitude towards your spouse, feelings of despair and frustration with your spouse, and your own failure [10–13].

There are no statistics on the rate of marital burnout in Iran, but the rising number of divorces in this country, has led researchers to conduct educational interventions to prevent the causes of divorce in order to prevent the growing trend of divorce in Iran [10]. Therefore, various educational interventions on marital burnout have been performed in Iran [3, 11, 14], but the special feature of this study is training using effective communication skills. The results of a systematic review study showed that interpersonal communication had a positive effect on marital satisfaction [15]. Communication skills are one of the best predictors of marital burnout. It seems that effective communication skills training can reduce the rate of marital burnout [16]. Couples who have effective communication skills express their desires more effectively, resolve their conflicts, share their thoughts and feelings more easily with each other, feel more intimacy and closeness to each other, and finally they experience a higher quality of marriage. A high quality of marriage helps couples to be less at risk of marital burnout [17, 18]. The results of a study showed that couples who had more effective communication skills had significantly less marital boredom and were less likely to divorce [19].

In the effective communication training intervention, teaching and practicing the skills of sending and receiving messages, conflict resolution, practicing speaking skills, active listening skills, focusing on the problem, recognizing stress, confirmation of differences, requesting feedback, appreciation, and responsibility [20]. The rate of marital burnout in women was reported higher than men [3, 21]. Studies have shown that the marital burnout has devastating effects and may lead to cause similar disorders in the children of these women [3, 21]. Also, women are more prone to stress due to the multiplicity of tasks they have, such as the upbringing of children, working at home, working outside the home, and as a result, they are more prone to marital burnout [22–24]. Therefore, women were considered as the study population and the present study was conducted to determine the effect of effective communication skills training intervention on the rate of marital burnout among married women referring to health centers.

The study's hypotheses were:

### Hypothesis 1

The experimental group and the control group have significant differences in marital burnout, and its dimensions include physical burnout, emotional burnout and psychological burnout.

### Hypothesis 2

In terms of effective communication skills, there are significant differences between the experimental group and the control group.

## Methods

This study consisted of a descriptive study and a quasi-experimental study. A descriptive study was conducted to determine the marital burnout status of 936 married women at the Gonabad Health Center in Iran [25]. In the second stage, a quasi-experimental study was conducted on women with high rates of marital burnout to determine the impact of effective communication skills training on marital burnout among married women in Gonabad, Iran in 2020.

### Inclusion and exclusion criteria

The inclusion criteria were: willing to participate in the study, filling out a written consent form, a resident of Gonabad, between the ages of 20 and 60, living with her husband and not suffering from mental illness. Exclusion criteria include: unwillingness to continue cooperation during the intervention program, absent more than one session in the training course, and divorce during the intervention program.

### Sample size

Based on the previous study [26] and considering the loss rate of 10%, the sample size of the intervention stage was calculated to be 94 participants (Experimental group = 47, Control group = 47).

### Sampling method

**Fig. 1:**
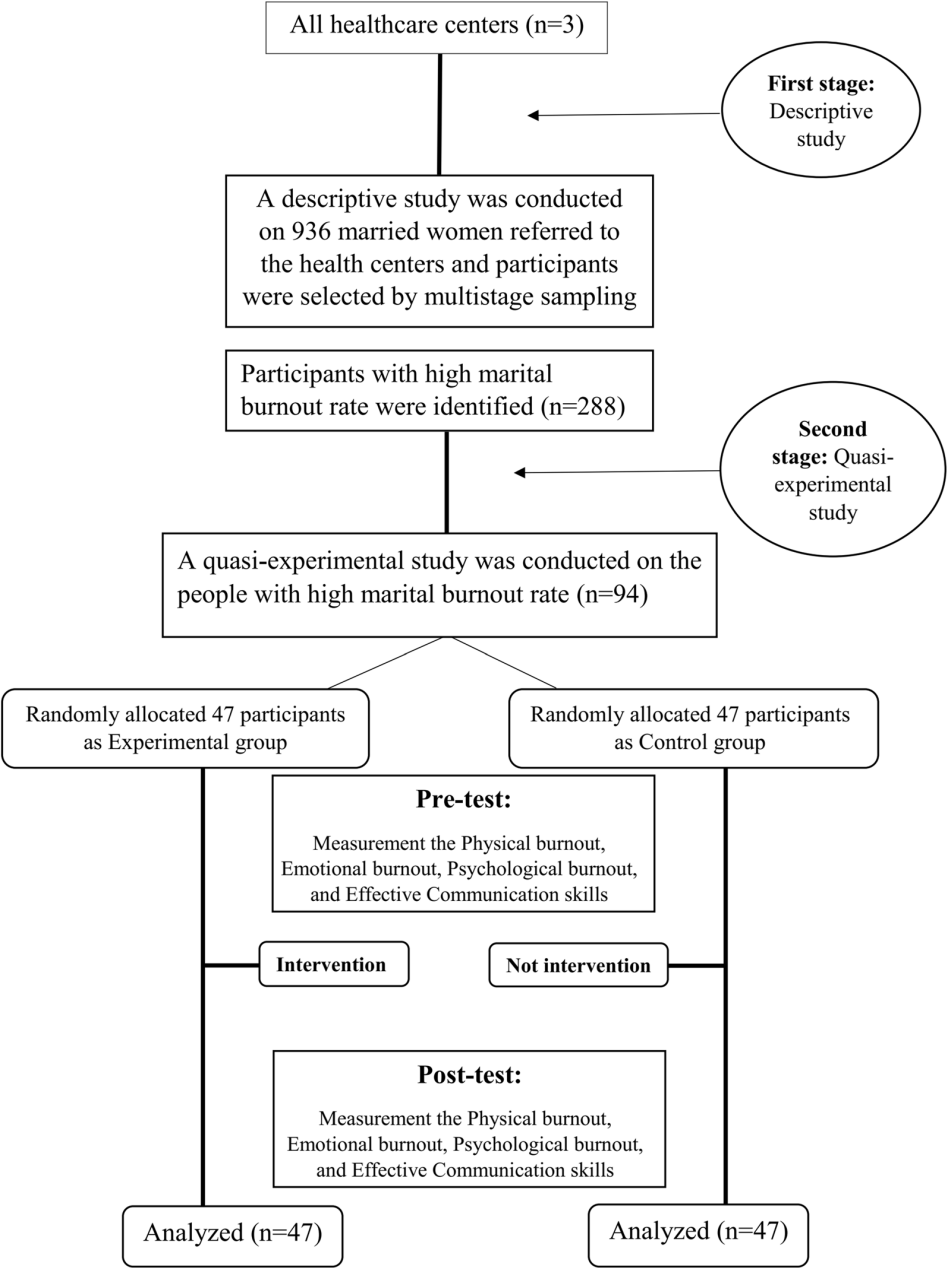
The flow of participants through each stage of the study

To select samples, 94 participants were selected from a descriptive study from among 936 married women referring to health centers who had a high rate of marital burnout and were randomly divided to the experimental group (n = 47) and control group (n = 47) (Fig. 1). The samples were randomly allocated to the control group and the experimental group by using NCSS PASS V.11 software.

### Ethical considerations

The research process began with obtaining the code of the ethics (IR.GMU.REC.1394.38) and registering in the clinical trial system (IRCT20180722040559N2). First, the objectives of the study were explained to the subjects and after obtaining informed consent from participants, questionnaires were given to them and was completed by self-report. The right to leave the study was reserved for those who did not wish to cooperate. Individuals are also assured that their information will be kept confidential from the research team.

### Data collection tools

In this study, three questionnaires were used for data collection, including the demographic section, the marital burnout questionnaire and the effective communication skills questionnaire.

#### The demographic questionnaire

This questionnaire included questions such as age, education level, occupation status, husband's education, husband's occupation, length of marriage, number children, marital satisfaction and etc. (Additional File 1).

#### Burnout measure (BM) scale

To measure the marital burnout of married women, the Pains BM Scale has been used [8]. This scale includes 21 questions and three subscales of Emotional burnout (feeling depressed, being emotionally exhausted, feeling hopeless, feeling anxious, feeling burned out, being troubled, and feeling worthless), Physical burnout (being tired, being physically exhausted, feeling energetic, being wiped out, being weary, feeling rundown, and feeling weak), and Psychological burnout (having a good day, feeling trapped, being happy, feeling disillusioned and resentful about people, feeling unhappy, feeling rejected, and feeling optimistic). Each subscale consists of 7 questions that are measured on a 7-point Likert scale (never, once in a long time, rarely, sometimes, usually, often, always) and in each subscale, the lowest score is 7 and the highest score is 49. The total marital burnout rate is based on the total score of each subscale. The lowest score is 21 and the highest score is 147 [8, 27]. In this study, the lower mean score indicates the lower degree of the couple burnout. The validity and reliability of this questionnaire in Iran have been examined and its Cronbach's alpha level has been reported to be 0.82 [28].

#### Effective communication skills questionnaire

This questionnaire was designed by Miller et al. and contains 25 questions about effective communication skills [29]. Questions were evaluated on a 6-point Likert scale (rarely = 1 to always = 6). Scoring some questions is the reverse. In this tool, the minimum score was 25 and the maximum score was 150. On this scale, a higher mean score indicates an improvement in effective communication skills. The validity and reliability of this questionnaire were examined in the Tavakolizadeh study and the Cronbach's alpha level was equal to 0.720 [30]. In this study, Cronbach's alpha of the effective communication questionnaire was 0.827.

### Educational intervention program

In the present study, an educational intervention was designed and performed in 7 training sessions of 45 min for the experimental group. Details of each training session were provided in Table 1. The questionnaires used in this study were assessed before the intervention and immediately after the intervention.

**Table 1:** Plan for conduction of the intervention

Sessions	Behavior change techniques	Intervention strategies
Session 1	Lecture, slides(PowerPoint)	Introducing the participants and outlining the goals and objectives of the training program In this session, we defining effective communication skills and explained the importance of this skills in marriage
Session 2	Group discussion and brainstorming, lecture, slides (PowerPoint)	In this session, we discussed the concept of effective communication skills, its importance and role in the marital relationship
Session 3	Group discussion and brainstorming, lecture, slides(PowerPoint)	In this session, we discussed the main elements of communication skills (verbal and non-verbal)
Session 4	Lecture, Group discussion, Pamphlets, slides(PowerPoint)	In this session, we discussed the types of communication behavioral styles ( passive, aggressive, passive-aggressive,, and assertive)
Session 5	Group discussion and brainstorming, lecture, slides(PowerPoint)	In this session, we discussed the active listening training, its necessity and functions, a variety of effective methods for active listening
Session 6	Group discussion and brainstorming, lecture, slides(PowerPoint),	In this session, we discussed the barriers to effective communication (such as humiliation, blame, defensiveness)
Session 7	Lecture, Group discussion	Summary of educational content, remove ambiguities and answer the participants' questions

### Data analysis

After entering the data into SPSS software version 24, first, the normality of the data was checked by the Kolmogorov–Smirnov test. Paired sample t-test and Wilcoxon test were used to compare the mean/median difference of the subscales before and after the intervention. To compare the mean/median of the subscales between the two groups before and after the intervention, t-test and Mann–Whitney tests were used. In all tests, the significance level was considered as *p* value less than 0.05.

## Results

In this study, the mean (standard deviation) age of participants was 36.39 (± 10.95). The majority of women (79.6%, n = 74) and their husbands (68.1%, n = 64) had diploma and under diploma. Approximately 81% (n = 76) of women were housewives and most of their husbands (61.7%, n = 58) were self-employed. Also, 8.5% (n = 8) of participants reported that they got married by family force. Other demographic information can be seen in Table 2.

**Table 2:** Socio-demographic characteristics of participants in baseline

Variable		Experimental group		Control group		All	*p* value
		N	%	N	%	N (%)	
Education	Diploma and under diploma	39	84.8	35	74.5	74 (79.6)	0.304*
	Academic	7	15.2	12	25.5	19 (20.4)	
Husband's education	Diploma and under diploma	31	66	33	70.2	64 (68.1)	0.825*
	Academic	16	34	14	29.8	30 (31.9)	
Occupation	Housewife	42	89.4	34	72.3	76 (80.9)	0.065*
	Employee	5	10.6	13	27.7	18 (19.1)	
Husband's occupation	Employee	19	40.4	17	36.2	36 (38.3)	0.832*
	Self- employee	28	48.3	30	63.8	58 (61.7)	
Meet the husband before the marriage	Yes	16	35.6	18	38.3	34 (37)	0.931*
	No	29	64.4	29	61.7	58 (63)	
Length of marriage	< 20	36	76.6	32	69.6	68 (73.1)	0.490*
	> 20	11	23.4	14	30.4	25 (26.9)	
Married by family force	Yes	4	8.5	4	8.5	8 (8.5)	0.990*
	No	43	91.5	43	91.5	86 (91.5)	
Your relationship with your spouse	Relative	20	42.6	16	34	36 (38.3)	0.525*
	Non-relative	27	57.4	31	66	58 (61.7)	
Engage in effective communication training courses	Yes	8	17	5	10.6	13 (13.8)	0.552*
	No	39	83	42	89.4	81 (86.2)	
Marital satisfaction	Yes	41	89.1	44	93.6	85 (91.4)	0.486*
	No	5	10.9	3	6.4	8 (8.6)	
Number of children	Does not have	0	0	2	4.7	2 (2.3)	0.187*
	1–2	30	68.2	25	58.1	55 (63.2)	
	3 and more	14	31.8	16	37.2	30 (34.5)	
Age	Mean (SD)	36.72	11.60	36.06	10.37	36.39 (10.95)	0.772**

*Chi-square, **Independent Samples T-test

The results of the chi-square and independent samples t-test showed that there was no significant difference in terms of demographic variables between the experimental and control groups before the intervention (*p* > 0.05) and the two groups were homogeneous at the beginning of the study (Table 2). Also, before the intervention, the mean/ median score of total marital burnout, subscales of physical burnout, emotional burnout, psychological burnout, and effective communication skills did not show a significant relationship between the experimental and control groups (*p* > 0.05) and two groups were homogeneous in terms of these variables (Table 3).

**Table 3:** Comparison of the mean and the median of constructs of couple burnout and effective communication

Variable	Group	Before intervention		After intervention		95% confidence interval		*p* value*
		Mean/median	SD/IQR	Mean/median	SD/IQR	Lower	Upper	
Physical burnout	Experimental	21.51	6.20	17.85	4.75	2.495	4.823	< 0.001
	Control	21.19	7.06	22.00	6.51	− 1.236	− 0.040	0.037
	*p* value**	0.817		0.001		–		
	(t)	0.233		− 3.382				
Emotional burnout	Experimental	19.27	6.17	16.00	4.72	2.309	4.244	< 0.001
	Control	17.21	6.46	18.40	6.28	− 1.858	− 0.524	0.001
	*p* value**	0.117		0.039		–		
	(t)	1.582		− 2.096				
Total marital burnout	Experimental	60.51	14.96	51.82	11.90	6.343	11.017	< 0.001
	Control	57.40	17.26	60.17	16.46	− 4.135	− 1.396	< 0.001
	*p* value**	0.354		0.006		–		
	(t)	0.932		− 2.814				
Effective communication skills	Experimental	85.12	15.86	97.95	14.53	− 15.766	− 9.893	< 0.001
	Control	85.91	13.71	85.95	12.47	− 0.852	0.767	0.916
	*p* value**	0.797		< 0.001		–		
	(t)	− 0.257		4.296				
Psychological burnout	Experimental	18	7 (16–23)	17	6 (15–21)	0	0.031	0.001****
	Control	19	6 (15–21)	20	7 (16–23)	0	0.031	0.002****
	*p* value***	0.641		0.053		–		
	(Z)	− 0.466		− 1.933				

IQR: Interquartile range

*Paired Samples T-Test, **Independent Samples T-test, ***Mann–Whitney Test, ****Wilcoxon test

After the intervention program, the results of paired sample t-test showed that the mean score of total marital burnout was significantly difference between two groups. This means that the educational intervention program significantly reduced the rate of total marital burnout in the experimental group (*p* < 0.001), while in the control group without any intervention, the rate of total marital burnout significantly increased (*p* < 0.001) (Table 3).

Based on the results of paired sample t-test, after the intervention program the mean score of the physical burnout subscale were difference between two groups. This means that the training program has significantly reduced the rate of physical burnout in the experimental group (*p* < 0.001) and in the control group who did not receive any training, the rate of physical burnout significantly increased (*p* < 0.05). The results of paired t-test after the intervention program showed that there was a significantly difference between two groups in term of emotional burnout subscale. This means that the training program significantly reduced the emotional burnout rate of the intervention group (*p* < 0.001), while in the control group that did not receive any intervention, the emotional burnout rate was significantly increased (*p* < 0.05) (Table 3).

In Table 3, after the intervention program the results of the Wilcoxon test showed that the median score of psychological burnout subscale were difference between two groups. This means that the educational intervention significantly reduced the rate of psychological burnout in the experimental group (*p* < 0.05), while in the control group without any intervention, the rate of psychological burnout significantly increased (*p* < 0.05) (Table 3). Based on the results of paired t-test, the mean score of effective communication skills after the intervention showed a significantly difference between two groups. This means that the rate of effective communication skills was significantly increased in the experimental group than control group (*p* < 0.001) (Table 3).

## Discussion

The results of this study showed that the mean/ median score of total marital burnout, subscales of physical burnout, emotional burnout, and psychological burnout decreased significantly in the experimental group compared to the control group. Also, the mean score of effective communication skills increased significantly in the intervention group compared to the control group.

The results of the this study showed that the mean score of total marital burnout after the intervention was significantly difference between two groups and the rate of total marital burnout was significantly decreased in experimental group. The results of Rajani's study showed that the implementation of cognitive-behavioral couple therapy in the experimental group significantly reduced conflict and marital burnout among couples [2]. A study conducted by Sirin with the aim of investigating the effect of family education programs on the marital burnout among married women showed that the implementation of this intervention program significantly reduced marital burnout among married women in the experimental group compared to the control group [31]. The results of Padash's study showed that educational intervention based on the couple therapy causes a significant reduction in marital burnout in the experimental group and also reduces the rate of divorce among married women [32]. The results of a study showed that solution-focused therapy intervention reduced the rate of marital burnout in the experimental group and improved their marital quality [33]. Implementing pre-marital or post-marital education programs has a positive effect on the quality of life and marital satisfaction of couples and reduces their marital burnout [34–36].

Based on the results of this study, after the intervention program there was a significant difference between two groups in term of the mean score of the physical burnout subscale and the rate of physical burnout reduced in the experimental group. The results of a study showed that training through concentrated group discussion significantly reduced the physical burnout of the experimental group of women whose husbands were not addicted [26]. The results of a similar study showed that the implementation of educational programs can significantly reduce the rate of physical burnout in women [37]. The results of a study showed that life skills training significantly reduced physical burnout of the experimental group [38]. A study conducted by Ahrari showed that communication skills training can significantly reduce marital conflicts and reduce physical burnout between couples with marital problems [39]. Marital conflicts and lack of a proper relationship between couples can led to various marital problems and reduce their quality of life. Over time these problems can lead to physical burnout, such as fatigue, boredom, headaches, etc. in couples [40].

In this study, after the intervention there was a significant difference in the mean score of the emotional burnout subscale between two groups and the mean score in the experimental group was significantly decreased compared to the control group. The results of an interventional study showed that the implementation of an educational intervention program significantly reduced women's emotional burnout in the experimental group [26]. Based on the results of an educational intervention study that was conducted to investigate the effect of emotional therapy on marital burnout, showed that the emotional burnout rate of women in the experimental group has been significantly reduced [37]. The results of a study, which was conducted to investigate the effect of emotionally-focused couple therapy, and showed that this intervention program significantly reduced the marital burnout in the experimental group compared to the control group [41]. The results of Allan's research showed that the implementation of emotion-focused couple therapy intervention reduces the effects of marital burnout and causes less harm to children [42]. Strengthening the emotional burnout among couples reduces their mental disorders, increases sexual satisfaction, marital adjustment, and ultimately increases the quality and marital satisfaction [43–45]. Having effective can establish a good emotional relationship between husband and wife, and prevent marital burnout and marital conflict [18, 39, 46].

In the present study, after the intervention, there was a significant difference in the median score of psychological burnout subscale between two groups, and the median score in the experimental group was significantly decreased compared to the control group. Based on the results of a similar study, the implementation of educational programs significantly reduced psychological burnout in the experimental group [26]. A study conducted by Asgari also showed that the implementation of an emotional schema therapy training program among couples who were going to divorce can reduced their psychological burnout and total marital burnout [14]. The results of Dehghan study showed that life skills training can improve women's life satisfaction, mental health and reduce marital burnout [47]. The results of a study showed that life skills training significantly reduced psychological burnout in the experimental group compared to the control group [38]. A study that was conducted to investigate the effect of communication skills on reducing marital conflict showed that strengthening these skills significantly reduced conflict and psychological burnout in the experimental group [40]. There is a relationship between mental health and marital burnout and marital satisfaction. People with better mental health status have higher marital satisfaction and less marital burnout [48–50].

The results obtained in this study showed that the mean score of effective communication skills after the intervention was significantly improved in the experimental group compared to the control group. Based on the results of Sajadi study, which was conducted to investigate the effect of effective communication skills training intervention on marital burnout status, showed that this training program significantly reduced marital burnout in the experimental group, and having communication skills is one of the effective factors in preventing marital burnout [51]. A study conducted by Tavakolizadeh showed that implementing a communication skills training program for married women in the experimental group can significantly reduce their marital conflicts [30]. Another study found that communication skills training significantly increased marital satisfaction and reduced marital conflict in the experimental group and improved the quality of life of couples [52]. The results of a study, which was conducted with the aim of enriching communication skills on reducing marital burnout, showed that strengthening these skills significantly reduces marital burnout and all aspects of burnout in couples and increases their marital quality [53]. When couples resolving their marital problems and conflicts, unsatisfied couples are more likely than other couples to show negative communicative behaviors, and rarely show positive communicative behaviors [54]. Having effective communication skills is one of the factors that reduce marital conflict, reduce the marital burnout, and improve marital quality between couples. Therefore, it is necessary to strengthen effective communication skills in premarital education programs to increase the quality of marital life of couples [55–58]. Therefore, women who believe that they do not have good communication skills and cannot solve marital problems can strengthen these skills with their husbands.

### Practice implications

Due to the positive impact of effective communication skills training in reducing marital burnout, these skills can be used as an important preventive factor in reducing marital problems. All couples are advised to participate in effective communication training courses with their spouses before getting married, so that they have less problems in their married life. Married couples are also encouraged to attend these classes if they do not have effective communication skills. To strengthen these skills, they can refer to psychologists and counselors who specialize in this field, or use the educational videos available in this field, or read related books in the field of communication skills. It is also necessary for counselors in premarital and family counseling programs to pay more attention to the positive role of effective communication and provide the necessary information to their clients in this regard.

### Strengths and limitations

One of the limitations of the present study was the lack of study on men and the study was performed only on women. Another limitation of the study was that the information was completed as a self-report before and after the intervention, which may be accompanied by a percentage of error. One of the limitations of this study was the lack of the survey of family income due to the lack of response from participants to this question. One of the advantages of the present study was the selection of participants based on a descriptive study that was selected for the intervention section from among these individuals. It is recommended that similar intervention plans for men and women be carried out in future studies.

## Conclusion

The results of the present study showed that the implementation of an effective communication skills training program can be effective in reducing total marital burnout and its aspects (subscales of physical, emotional and psychological burnout) in married women. Having effective communication skills makes it possible for women to resolve their marital conflicts instead of escaping from marital problems. Solving marital problems can reduce their marital burnout and improve their quality of life. Considering the positive impact of effective communication skills training on reducing marital burnout, it is recommended that counselors and psychologists pay more attention to enhancing these skills in premarital training programs and couple therapy courses. It is also recommended that effective communication skills be taught to student adolescents in schools, so that these people will have a higher quality of marriage in the future and ultimately have a higher quality of life.

## Supplementary Information

**Additional file 1**. Demographic Characteristics Questionnaire.

## Data Availability

The data sets used and/or analyzed during the current study was available from the corresponding author on reasonable request.
