# Supplementary material for: The impact of effective communication skills training on the status of marital burnout among married women
Source: BMC Womens Health. 2021 Jun 3;21:231. doi: 10.1186/s12905-021-01372-8 (PMC8176605; doi:10.1186/s12905-021-01372-8)
Supplement: Supplementary file 1 — Additional file 1. Demographic Characteristics Questionnaire. [file 12905_2021_1372_MOESM1_ESM.pdf]

# Demographic Characteristics Questionnaire

- **Group:** Experimental □ Control □
- **Age (Year)………………………**
- **Number of children ………………….**
- **You're Education level**

Illiterate □

Elementary school □

High school or Diploma □

Associate or Bachelor's degree □

Master's degree or High degree □

- **Husband's education level**

Illiterate □

Elementary school □

High school or Diploma □

Associate or Bachelor's degree □

Master's degree or High degree □

- **You're Occupation statues**

Housewife □

Employee □

Self- Employee □

Others……………….

- **Husband's occupation statues**

Employee □

Self- Employee □

Unemployed □

Labor □

Others……………….

- **Did you know your spouse before you got married?**

  Yes ☐

  No ☐

- **Length of your marriage………………**
- **Are you married by family force?**

  Yes ☐

  No ☐

- **What is your relationship with your spouse?**

  Relative ☐

  Non-relative ☐

- **Have you ever participated in effective communication skills training programs?**

  Yes ☐

  No ☐

- **Are you generally satisfied with your marriage?**

  Yes ☐

  No ☐
